# Oncolytic Viruses as a Novel Therapeutic Approach for Colorectal Cancer: Mechanisms, Current Advances, and Future Directions

**DOI:** 10.3390/cancers17111854

**Published:** 2025-05-31

**Authors:** Francisco Pérez-Domínguez, Claudia Quezada-Monrás, Leonardo Cárcamo, Juan P. Muñoz, Diego Carrillo-Beltrán

**Affiliations:** 1 Laboratorio Oncovirología Molecular, Instituto de Bioquímica y Microbiología, Facultad de Ciencias, Universidad Austral de Chile, Valdivia 5090000, Chile; francisco.perez.d@ug.uchile.cl; 2 Servicio Medicina Interna, Hospital Base Valdivia, Valdivia 5090000, Chile; 3 Laboratorio de Biología Tumoral, Instituto de Bioquímica y Microbiología, Facultad de Ciencias, Universidad Austral de Chile, Valdivia 5090000, Chile; claudiaquezada@uach.cl; 4 Millennium Institute on Immunology and Immunotherapy, Facultad de Ciencias, Universidad Austral de Chile, Valdivia 5110566, Chile; 5 Servicio de Cirugía, Hospital Base Valdivia, Valdivia 5090000, Chile; leonardo.carcamo.gruebler@uach.cl; 6 Laboratorio de Bioquímica, Departamento de Química, Facultad de Ciencias, Universidad de Tarapacá, Arica 1000007, Chile

**Keywords:** oncolytic viruses, colorectal cancer, immunotherapy, virotherapy, adenovirus

## Abstract

This review provides an updated overview of oncolytic virotherapy as a promising therapeutic strategy for colorectal cancer (CRC), focusing on six key viral platforms: adenovirus, herpes simplex virus (HSV), reovirus, vesicular stomatitis virus (VSV), vaccinia virus (VV), and measles virus (MV). These viruses exhibit tumor-selective replication and exert their effects through mechanisms such as direct oncolysis, the delivery of immunostimulatory genes (e.g., IL-12, IL-15, GM-CSF), the activation of innate and adaptive immune responses, and the remodeling of the tumor microenvironment. Preclinical and early clinical studies suggest that oncolytic viruses can enhance the efficacy of existing treatments, particularly in immunologically “cold” tumors such as microsatellite stable CRC, when used in combination with chemotherapy or immune checkpoint inhibitors. Despite encouraging results, several challenges remain, including antiviral immune clearance, tumor heterogeneity, and limitations in systemic delivery. Current research focuses on improving viral engineering, enhancing tumor targeting, and designing combinatorial strategies to overcome resistance and maximize clinical benefits. Overall, oncolytic viruses represent a versatile and evolving therapeutic class with the potential to address unmet clinical needs in CRC.

## Simple Summary

Colorectal cancer (CRC) remains one of the most prevalent and deadly malignancies worldwide, often diagnosed at advanced stages and showing limited response to conventional therapies. Oncolytic virotherapy has emerged as a novel and promising approach that combines direct tumor cell killing with immune system activation. This review summarizes the current state of oncolytic viruses (OVs) under investigation for CRC, including adenovirus, herpes simplex virus (HSV), reovirus, vesicular stomatitis virus (VSV), vaccinia virus (VV), and measles virus (MV). Each viral platform is discussed in terms of its mechanism of action, preclinical and clinical data, limitations, and future directions. Overall, oncolytic viruses represent a dynamic and evolving therapeutic class with the potential to address unmet needs in CRC treatment and improve patient outcomes.

## 1. Introduction

Colorectal cancer (CRC) was the fourth most commonly diagnosed cancer and the second leading cause of cancer-related deaths in the United States in 2024, following lung and bronchial cancer [1]. Due to widespread screening programs, CRC incidence has declined over recent decades. However, since the mid-1980s, incidence rates have been increasing among adults aged 20–39, with a similar upward trend observed in individuals aged 40–54 since the mid-1990s. Between 2011 and 2019, the incidence rate increased by approximately 2% annually in those under 50, including individuals aged 50–54 [2]. Interestingly, despite a significant annual decline of 4.3% in the incidence of localized-stage disease from 2006 to 2019, there has been an increase in advanced-stage diagnoses [2]. The transition to later-stage diagnoses can be attributed to the saturation of screening programs, the tendency to disproportionately detect and remove slower-growing adenomas rather than more aggressive ones, and the rising prevalence of early-onset CRC, which is more frequently diagnosed at an advanced stage [3]. The first-line treatment of advanced or metastatic CRC (mCRC) typically consists of a chemotherapy combination, often associated with a biologic agent, based on the specific molecular profile of the patient. For this reason, patients with advanced CRC should undergo genetic tumor analysis to detect KRAS/NRAS and BRAF mutations, HER2 amplifications, and microsatellite instability (MSI)/mismatch repair (MMR) status, among other biomarkers [4]. The chemotherapy drugs utilized in first- and second-line treatment for mCRC include 5-Fluorouracil (5-FU), capecitabine, oxaliplatin, and irinotecan, which may be combined with biologic agents targeting the vascular endothelial growth factor (VEGF) and epidermal growth factor receptor (EGFR) pathways, as well as immunotherapy options [5]. Bevacizumab is a humanized monoclonal antibody that inhibits VEGF, a key factor in tumor angiogenesis. Combined data from multiple randomized phase II trials have demonstrated that incorporating bevacizumab into first-line 5-FU therapy enhances overall survival (OS) in patients with unresectable mCRC compared to those treated with these regimens without bevacizumab [6,7]. Similarly, cetuximab and panitumumab, monoclonal antibodies targeting EGFR and blocking its downstream signaling pathways, offer significant clinical benefits in treating patients with RAS wild-type mCRC [8,9].

**Figure 1:**
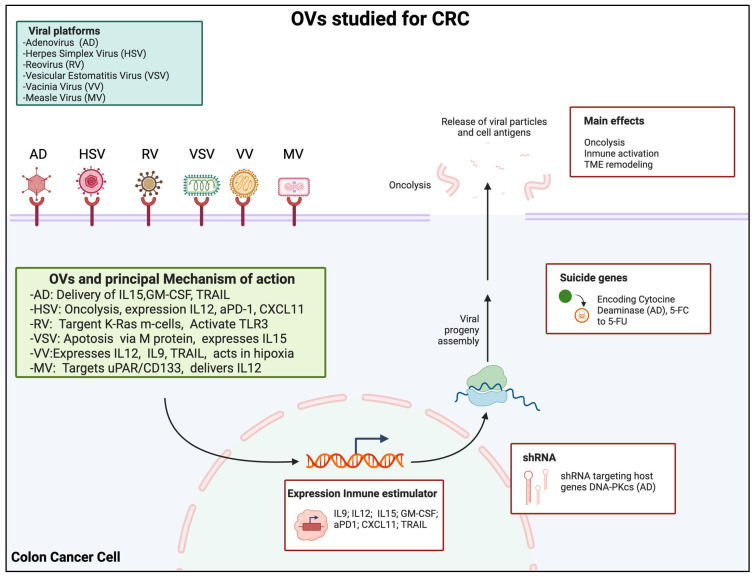
Schematic representation of the leading oncolytic virus platforms investigated for colorectal cancer (CRC) and their mechanisms of action. Adenoviruses (AD) selectively replicate in tumor cells and deliver therapeutic genes such as IL-15, GM-CSF, and TRAIL, promoting direct lysis and immune activation. Herpes simplex viruses (HSV) induce tumor cell death and express immunomodulatory molecules such as IL-12, CXCL11, and anti–PD–1 antibodies, facilitating CD8^+^ T cell recruitment and the remodeling of the tumor microenvironment (TME). Reoviruses preferentially infect KRAS-mutant cells and activate innate immunity via TLR3, leading to apoptotic tumor cell death. Vesicular stomatitis viruses (VSV) trigger apoptosis through their matrix protein and are armed with IL-15 to enhance CD8^+^ and NK cell responses while modulating interferon signaling. Vaccinia viruses (VV) act effectively in hypoxic tumor areas and express a range of cytokines, including IL-12, IL-9, IL-23, and TRAIL, contributing to immune cell infiltration and the suppression of tumor-promoting signals. Measles viruses (MV) target tumor-initiating cells expressing uPAR or CD133 and are often engineered to express IL-12, inducing durable anti-tumor immune responses and long-term memory. (Created in https://BioRender.com. Accessed on 13 May 2025).

Despite these advancements, several limitations have been identified in the existing first-line treatments. One of the most relevant challenges is the diverse molecular nature of colorectal tumors, which plays a crucial role in treatment resistance, prompting the exploration of combination therapies designed to bypass both intrinsic and acquired resistance by simultaneously targeting multiple carcinogenic pathways. However, a significant drawback of using numerous targeted agents is the increased risk of cumulative toxicity [10,11]. These limitations have driven the development of novel therapeutic approaches, among which virotherapy has emerged as a promising option. Oncolytic virotherapy refers to genetically engineered or naturally occurring viruses that selectively infect, replicate, and destroy cancer cells while sparing normal tissues. This selectivity is often attributed to the impaired antiviral responses and dysregulated signaling pathways within tumor cells [12]. Unlike traditional therapies such as chemotherapy and radiotherapy, which often lack specificity and can lead to systemic toxicity, oncolytic viruses (OVs) provide both direct tumor lysis and a robust activation of anti-tumor immune responses, offering a dual therapeutic effect [13]. Several viral platforms have been explored for colorectal cancer (CRC), including adenovirus, herpes simplex virus (HSV), reovirus, vesicular stomatitis virus (VSV), vaccinia virus (VV), and measles virus, each with distinct mechanisms of action, safety profiles, and clinical progress [14]. Given these properties, OVs represent a compelling and versatile strategy within the expanding arsenal of cancer immunotherapies (Figure 1) [15].

In this review, we describe the mechanisms of action of OVs, analyze the most recent preclinical and clinical studies in CRC, and discuss the limitations, challenges, and future perspectives in this field of study.

## 2. Oncolytic Viruses

OVs are self-replicating microorganisms, either naturally occurring or genetically modified, that selectively infect and multiply within tumor cells [16]. Their ability to preferentially target cancer cells arises from the molecular abnormalities that drive tumor progression, such as defects in antiviral signaling pathways and dysregulated cell cycle control [17]. Tumorigenesis is a multistep process involving gene mutations, which collectively lead to malignant transformation. OVs exploit these vulnerabilities, allowing for selective replication in tumor cells while sparing normal tissues.

The anti-oncogenic effects of OVs have been recognized since the mid-20th century, as shown in studies by Moore [18,19]. However, it was not until recent advances were made in molecular oncology that the development of OV-based therapies became feasible on a clinical scale [20]. A major breakthrough occurred with a Phase III randomized clinical trial evaluating a genetically engineered herpes simplex virus type 1 (HSV-1) expressing granulocyte–macrophage colony-stimulating factor (GM-CSF). This trial demonstrated a significantly higher durable response rate in patients with advanced melanoma, leading to the approval of this virus (talimogene laherparepvec (T-VEC)) by the U.S. Food and Drug Administration (FDA) as the first oncolytic virus-based immunotherapy for cancer [21].

OVs eliminate tumor cells through multiple mechanisms. First, they induce direct oncolysis by replicating within tumor cells, leading to cell lysis and the release of new viral particles that spread the infection. This process is facilitated by tumor-specific genetic mutations that allow for viral replication while restricting infection in healthy tissues [20]. Second, OVs trigger immunogenic cell death, releasing tumor-associated antigens (TAAs) and danger-associated molecular patterns (DAMPs), which stimulate anti-tumor immunity. This activation enhances dendritic cell function and primes cytotoxic T lymphocytes to target cancer cells beyond the initial infection site [20,22]. Third, OVs reshape the tumor microenvironment (TME) by counteracting immunosuppressive signals. They inhibit regulatory T cells and myeloid-derived suppressor cells while promoting the release of proinflammatory cytokines and chemokines that strengthen immune responses [23].

Additionally, some OVs are engineered to express therapeutic genes that enhance their anti-cancer efficacy. These modifications may include the expression of cytokines, prodrug-converting enzymes, or immune checkpoint inhibitors (ICIs) that further amplify immune activation [24,25]. Together, these mechanisms create a robust anti-tumor response, positioning OVs as promising agents in cancer therapy, particularly in combination with immunotherapies and conventional treatments.

Among emerging cancer treatments, combining oncolytic virotherapy with other immunotherapies—particularly ICIs and chimeric antigen receptor T-cell (CAR-T) therapy—has shown significant potential [26,27]. OVs play a crucial role in converting immunologically “cold” tumors into “hot” ones, thereby increasing their sensitivity to ICIs [28]. Additionally, OVs can function as carriers for CAR-T cells, facilitating their infiltration into the tumor and overcoming the immunosuppressive microenvironment, thus enhancing their cytotoxic activity against solid tumors [29]. This dual approach capitalizes on the immune-modulating properties of OVs to improve the efficacy of ICIs and CAR-T therapies, making it a promising strategy in cancer treatment.

## 3. Types of OVs Studied for CRC

### 3.1. Adenovirus

#### 3.1.1. Mechanism of Action

Adenoviruses are non-enveloped, double-stranded DNA viruses with a well-characterized genome, making them highly amenable to genetic engineering [30,31]. Their high transduction efficiency allows for effective gene delivery to various cell types. Additionally, their ability to induce strong immune responses makes them valuable for vaccine development and oncolytic therapy [32].

Oncolytic adenoviruses exhibit selective replication in CRC cells, leading to the direct lysis of tumor cells and the release of tumor-associated antigens [33]. Gene-silencing strategies have also been explored, such as an shRNA-expressing adenovirus targeting DNA-PKcs, a key enzyme in DNA repair. Adenoviral vectors have modulated the TME, facilitating systemic anti-tumor effects such as the abscopal phenomenon [34]. The telomerase-specific oncolytic adenovirus OBP-301 (Telomelysin) utilizes extracellular vesicles (EVs) to deliver viral components to distant metastatic sites [35]. These EVs induce apoptosis and autophagy while circumventing immune suppression, thereby increasing tumor-specific targeting and reducing off-target effects [36]. This mechanism supports combination with ICIs for enhanced immunotherapeutic outcomes [36]. Genetic engineering has enabled the expression of immune-stimulatory molecules by oncolytic adenoviruses, amplifying their therapeutic impact. A prominent example is the development of IL-15-expressing adenoviruses, which enhance the activity of natural killer (NK) cells, CD8^+^ T cells, and other immune effectors [37,38]. In CRC models, combining IL-15-expressing oncolytic adenoviruses with cytotoxic T lymphocytes (CTLs) has demonstrated superior tumor suppression, reinforcing the potential of virotherapy in immuno-oncology [37]. Another innovation is the construction of a bicistronic oncolytic adenovirus (Ad-CD-GMCSF), encoding cytosine deaminase (CD) and granulocyte-macrophage colony-stimulating factor (GM-CSF). CD converts the prodrug 5-fluorocytosine (5-FC) into the chemotherapeutic agent 5-FU, inducing tumor cell death, while GM-CSF recruits antigen-presenting cells, such as dendritic cells and macrophages, to enhance T-cell priming [39,40]. This dual-function approach improved tumor clearance, enhanced survival, and modulated the TME by reducing immunosuppressive cytokines [39].

#### 3.1.2. Clinical and Preclinical Evidence

Studies demonstrate improved efficacy via co-administration with chemotherapy or gene silencing vectors. Several adenoviral constructs show tumor suppression in xenograft models and patient-derived xenografts. This oncolytic activity promotes T-cell infiltration and activation, thereby enhancing the efficacy of ICIs [41]. In the same way, enhancements such as L-carnosine loading improve viral transduction and apoptosis, boosting therapeutic efficacy [42]. A notable example is the chimpanzee-derived adenovirus AdC7-SP/E1A-ΔE3, which is engineered to bypass pre-existing immunity to human adenoviruses. This construct selectively replicates in CRC cells and induces p53-independent mitochondrial apoptosis [43]. Further strategies involve combination therapies to enhance efficacy. For instance, a conditionally replicative adenovirus (CRAd) co-administered with valproic acid (VPA), a histone deacetylase inhibitor, significantly increased tumor suppression relative to either agent alone. Mechanistically, this combination reduced proliferation, triggered DNA damage, increased polyploidy, and induced H2AX phosphorylation, which is indicative of DNA double-strand breaks, while downregulating DNA repair proteins [44].

In adenovirus gene-silencing strategies, this vector increased radiosensitivity; its effect was limited in xenograft models [45,46,47,48]. However, combining it with a CRAd improved gene delivery and radiosensitization, underscoring the promise of adenovirus-based radiosensitizing approaches for CRC [48]. Another study designed a conditionally replicative oncolytic adenovirus, Ad312-E1A, to target CRC cells with IGF2 imprinting (LOI) loss, a common epigenetic abnormality in CRC and other malignancies [49,50]. The virus selectively replicates in IGF2 LOI-positive tumor cells, reducing cell viability and inducing apoptosis, while sparing normal cells. In xenograft models, Ad312-E1A effectively suppressed tumor growth and prolonged survival, demonstrating its potential as a targeted gene therapy approach [50]. Another strategy involved an adenovirus engineered to deliver TRAIL (tumor-necrosis-factor-related apoptosis-inducing ligand), which induced apoptosis with minimal impact on healthy cells. In patient-derived xenograft models, the TRAIL-expressing construct (Ad/TRAIL-E1) provided superior tumor suppression and survival benefits compared to conventional virotherapy [51].

Another telomerase-specific oncolytic adenovirus, OBP-502, enhances ICI efficacy by promoting immunogenic cell death (ICD). It induces the release of ATP and HMGB1, leading to CD8+ T-cell infiltration and Foxp3+ T-cell suppression, thereby improving anti-tumor immunity. When combined with PD-1 blockade, OBP-502 exhibits synergistic effects, including tumor suppression and an abscopal effect in CRC models [52]. Furthermore, a synergy between oncolytic adenovirus dl1520 (ONYX-015) and chemotherapy (melphalan) has demonstrated enhanced tumor cell death in colorectal adenocarcinoma models [53]. ONYX-015 selectively replicates in p53-deficient tumors, constituting over 50% of all cancers, making it a promising targeted therapy [54]. Combining ONYX-015 with chemotherapy significantly increases apoptosis and inhibits proliferation, highlighting the potential of virus–chemotherapy combinations [53]. These findings suggest that oncolytic virotherapy can enhance conventional treatments, improving cancer cell selectivity and reducing resistance.

### 3.2. Herpes Simplex Virus (HSV)

#### 3.2.1. Mechanism of Action

HSV is a large double-stranded DNA virus with a 152 kb genome enclosed in an icosahedral capsid and a lipid envelope [55]. These structural and genomic features make HSV an attractive platform for genetic modification to improve tumor specificity and patient safety. Unlike retroviruses, HSV replicates within the host cell nucleus without integrating into the host genome, thus avoiding insertional mutagenesis. Furthermore, the virus remains sensitive to standard antiviral agents, adding a layer of safety for clinical use [56]. Oncolytic HSVs (oHSVs) selectively replicate in tumor cells, promoting direct lysis and the release of tumor-associated antigens. This cytolytic activity triggers immunogenic cell death and activates innate and adaptive immune responses. Additionally, oHSVs can be engineered to express immunostimulatory genes, further enhancing anti-tumor immunity and therapeutic efficacy [57,58].

In summary, oHSVs selectively replicate in tumor cells, causing oncolysis and antigen release. They modulate the tumor microenvironment and can be engineered to express immunostimulatory transgenes.

#### 3.2.2. Clinical and Preclinical Evidence

One of the primary therapeutic mechanisms of oHSV is direct tumor cell lysis. NV1020 is a well-studied oHSV that selectively replicates in cancer cells, inducing cell death while sparing normal tissue [59,60]. Preclinical studies have shown that NV1020, when combined with chemotherapeutic agents such as 5-FU, SN38, or oxaliplatin, leads to enhanced tumor suppression through additive or synergistic interactions. In murine CRC models, this combination therapy significantly reduced tumor volume and prolonged survival [61]. Another notable example is NV1066, an oHSV variant capable of selectively targeting and eliminating tumor-initiating cells (TICs) in CRC. TICs, identified through serum-free culture, exhibit high tumorigenic potential and overexpress Akt1, a protein linked to cancer cell survival and stemness [62,63]. NV1066 efficiently infects and replicates within TICs, inducing oncolysis and reducing tumor growth in vivo [63]. Targeting cancer stem-like cells (CSCs) further, Signal-Smart 2 (SS2), an engineered oHSV-1, was developed to selectively eliminate CD133^+^ cells, a subpopulation associated with tumor aggressiveness and therapeutic resistance. SS2 exhibited high specificity and reduced tumor invasiveness and progression in both in vitro and in vivo models [64]. Similarly, oHSV-2 has demonstrated potent anti-tumor effects by selectively targeting CSCs and bulk tumor cells. In CRC models, oHSV-2 treatment resulted in tumor necrosis and reduced cell invasion, and it synergized with 5-FU to enhance therapeutic efficacy [65,66].

One approach by which OHVs transform the TME involves High-Mobility Group Box 1 (HMGB1), which is a ubiquitous and highly conserved protein involved in gene regulation, immune response, and apoptosis [67,68]. Previous studies suggest that HMGB1 can inhibit aerobic respiration and alter mitochondrial metabolism, potentially leading to tumor cell death under low-oxygen conditions [69,70]. Hence, oHSV-1 expressing the HMGB1 protein (HSV-HMGB1) has shown enhanced cytotoxicity in normoxia but, paradoxically, increased colon cancer cell viability in hypoxia by inducing autophagy rather than apoptosis. A mechanistic analysis revealed that HSV-HMGB1 modulates the subcellular localization of HMGB1 and p53, influencing cell survival pathways [71]. Likewise, oHSV2 effectively targets murine colon carcinoma by altering the immune microenvironment and inducing anti-tumor immunity [72]. The virus reduces immunosuppressive cells while increasing CD8+ T cells, NK cells, and dendritic cells, thereby enhancing systemic immune responses. In vivo, oHSV-2 treatment eradicated primary tumors, prevented recurrence, and improved survival without notable toxicity, underscoring its potential as a safe and effective therapeutic agent [72].

oHSVs can be further modified to express immunostimulatory molecules, amplifying host anti-tumor immunity. One such construct, oHSV1-IL15B, encodes the IL-15/IL-15Rα complex to enhance cytotoxic T-cell responses. Combined with oHSV1-aPD1 expressing an anti-PD-1 antibody, this dual therapy elicited robust CD8^+^ T-cell activation and increased tumor apoptosis [73,74,75]. Another attenuated HSV-1 vector, HSV-G47Δ, has shown efficacy in an orthotopic rectal cancer mouse model by reducing tumor size and improving survival without systemic toxicity. The study also revealed that inhibition of the protein kinase R (PKR) antiviral pathway enhanced viral replication and oncolytic activity [76]. Further advancements include the development of O-HSV1211, an oHSV-1 engineered via CRISPR/Cas9 to express IL-12 and the chemokine CXCL11. This construct promoted immune cell infiltration, stimulated interferon-gamma (IFN-γ) production, and significantly improved tumor control and survival in CRC models [77].

### 3.3. Reovirus

#### 3.3.1. Mechanism of Action

Reovirus is a non-enveloped, double-stranded RNA virus with a segmented genome and an icosahedral capsid [78]. It selectively replicates in cancer cells harboring activated Ras signaling pathways, leading to direct tumor cell lysis and the activation of anti-tumor immune responses [79]. In addition to inducing innate and adaptive immunity, reovirus enhances T-cell and NK cell activity. Its capacity for systemic delivery and compatibility with chemotherapy and immunotherapy strategies highlights its potential as a promising oncolytic platform [80]. The following sections summarize the latest findings regarding the mechanisms of action of reoviruses in colon tumors.

One of the principal mechanisms of reovirus-mediated oncolysis involves modulating intracellular signaling pathways. Glycogen synthase kinase-3β (GSK-3β), a central regulator of the Wnt/β-catenin signaling pathway, is known to phosphorylate β-catenin, thereby promoting its degradation and attenuating oncogenic signaling [81]. The inhibition of GSK-3β has been shown to enhance reovirus-induced apoptosis in colon cancer cells. Although the upregulation of β-catenin does not increase viral replication, GSK-3β inhibition suppresses NF-κB activity, accelerating apoptosis via caspase-8 activation. This co-treatment strategy significantly amplifies cancer cell death compared to reovirus alone [82]. Another signaling pathway that has been evaluated is KRAS, a proto-oncogene that encodes a small GTPase involved in cell signaling pathways that regulate tumor growth, differentiation, and survival [83]. Reovirus can also reshape the TME, enhancing immune surveillance and systemic anti-tumor effects [84].

Reovirus has been shown to directly stimulate immune effector cells, particularly NK cells. Viral exposure increases NK cell cytotoxicity in a dose-dependent manner by activating Toll-like receptor 3 (TLR3) signaling. This activation leads to the upregulation of cytotoxic proteins such as perforin and granzymes, along with proinflammatory cytokines including TNF-α and IFN-γ [85]. Furthermore, when reovirus is combined with cetuximab, an anti-EGFR monoclonal antibody, it significantly enhances antibody-dependent cell-mediated cytotoxicity (ADCC) against CRC cells, regardless of the KRAS mutation status. Compared to monotherapies, this combinatorial approach leads to tumor suppression in preclinical models [86].

Another immune-enhancing strategy involves inhibiting transforming growth factor-beta (TGF-β), a cytokine that promotes tumor progression by facilitating epithelial-to-mesenchymal transition and immune evasion. TGF-β blockade significantly boosts reovirus-induced T-cell infiltration. Combined with CD3-bispecific antibody therapy, this regimen achieves complete tumor regression in CRC models [87,88].

In summary, reovirus preferentially targets KRAS-mutant cells, inducing oncolysis and activating innate immune pathways, particularly via TLR3.

#### 3.3.2. Clinical and Preclinical Evidence

Oncolytic reovirus preferentially induces apoptosis in KRAS-mutant CRC cells and synergizes with irinotecan, a topoisomerase I inhibitor [89]. The oral administration of reovirus RC402 has also been tested, which activates immune responses in Peyer’s patches of the terminal ileum. This stimulation promotes antigen presentation and T-cell activation in distant tumor sites, even without direct viral infection of the tumors. Notably, this effect depends on the gut microbiome, as its depletion abolishes the therapeutic benefit. When combined with immune checkpoint inhibitors (anti–PD-1 and anti–CTLA-4), oral reovirus administration achieves complete tumor regression and establishes durable immune memory, highlighting its promise as a non-invasive immunotherapy [90]. Likewise, Augustine et al. (2022) [84] have shown that oncolytic reovirus enhances immune responses in microsatellite-stable CRC (MSS CRC), making it more sensitive to anti-PD-1 therapy. Reovirus induces direct tumor cell apoptosis, activates innate immune pathways, increases PD-L1 expression, and promotes T-cell infiltration, reducing immunosuppressive macrophages. In murine models, the combination of reovirus and anti-PD-1 significantly reduces tumor growth and improves survival, which suggests that reovirus can convert immune-resistant MSS tumors into immunotherapy-responsive cancers [84]. In a clinical setting, reovirus administration in metastatic CRC patients harboring KRAS mutations has demonstrated substantial immunomodulatory effects. Treatment increased anti-tumor cytokines (GM-CSF, IL-12, IFN-γ), decreased pro-tumorigenic cytokines (IL-8, VEGF), and activated antigen-presenting cells and CD8^+^ T cells. In addition, reovirus suppressed the expression of miR-29a-3p, a microRNA associated with CRC progression. Transcriptomic profiling revealed an upregulation of immune-related gene expression, reinforcing the dual role of reovirus as both a cytolytic agent and an immune stimulator [91].

### 3.4. Vesicular Stomatitis Virus (VSV)

#### 3.4.1. Mechanism of Action

VSV is an RNA virus with a bullet-shaped morphology, a rapid replication cycle, and broad cellular tropism, making it an ideal oncolytic virus [92]. VSV selectively infects and kills cancer cells with defective IFN responses, while sparing normal cells. It induces direct oncolysis and triggers immune responses, enhancing anti-tumor immunity [93]. Due to its low pre-existing immunity in humans, ease of genetic modification, and ability to deliver therapeutic transgenes, VSV is a promising candidate for oncolytic virotherapy in CRC [94]. A key mechanism of VSV-mediated tumor suppression is the induction of apoptosis via its matrix (M) protein. Mutant forms of the M protein, such as ΔM51 and ΔM51R, have been studied for their improved safety and efficacy profiles. Both variants have demonstrated the ability to trigger caspase-dependent apoptosis in CRC cells, with ΔM51 offering enhanced biosafety due to a lower risk of reversion to a pathogenic phenotype [95]. VSV also exerts immunomodulatory effects that reshape the TME.

#### 3.4.2. Clinical and Preclinical Evidence

In a murine model of the peritoneal surface dissemination (PSD) of CRC, the intraperitoneal administration of ΔM51R VSV significantly inhibited tumor progression, prolonged survival, and reprogrammed the TME. Notably, this intervention increased CD4^+^ T cells and peritoneal immune cell populations, while reducing immunosuppressive MDSCs and proinflammatory cytokines such as IL-6 and MCP-1 [96]. To further enhance immunotherapeutic efficacy, VSV has been genetically engineered to express interleukin-15 (IL-15), a potent cytokine that stimulates NK cells and CD8^+^ cytotoxic T cells. In murine models of metastatic colon adenocarcinoma, localized IL-15 expression from VSV significantly improved survival and promoted tumor clearance [97,98]. This effect was notably superior to systemic IL-15 administration, emphasizing the therapeutic potential of oncolytic viruses armed with immune-stimulating transgenes [98]. VSV is a promising cancer therapy; however, antiviral immune responses must be considered [99,100]. To address this limitation, Alluqmani et al. evaluated the combination of VSVΔ51 with the immunomodulatory enhancer vanadyl sulfate (VS) in colon cancer models, demonstrating that VS enhances viral replication and shifts immune signaling from type I to type II interferon responses [101,102]. The combined therapy significantly increased proinflammatory cytokine secretion, improved tumor antigen-specific T-cell responses, and enhanced overall anti-tumor immunity [101]. Similarly, octyl itaconate (4-OI) is a macrophage inflammatory response suppressor that modulates the transcriptional regulator NRF2 by alkylating its repressor KEAP1, thereby reducing inflammation and inhibiting type I IFN responses [103,104]. When used with VSVΔ51, 4-OI significantly enhanced viral replication and oncolysis, particularly in IFN-resistant colon cancer cells. Mechanistic studies showed that 4-OI alters critical components of the MAVS, NF-κB, and IFN pathways, resulting in improved therapeutic outcomes. Both in vitro and in vivo experiments, including studies on patient-derived tumoroids, confirmed the increased efficacy of this combination in resistant CRC models [105].

### 3.5. Vaccinia Virus (VV)

#### 3.5.1. Mechanism of Action

VV is a large double-stranded DNA virus that replicates in the cytoplasm, avoiding host genome integration, making it a safe and stable oncolytic agent [106]. It has natural tumor tropism and a rapid replication cycle, and it can infect cancer cells under hypoxic conditions, which are common in tumors. Its large genome (~190 kb) allows for genetic modifications to express therapeutic genes (e.g., cytokines, immune checkpoint inhibitors), boosting anti-tumor immunity [107]. These properties make VV a versatile and practical oncolytic virus, capable of direct oncolysis, vascular disruption, and immune activation, improving its potential for combination cancer therapies. VV infection has also been shown to increase the infiltration of iNOS^+^ myeloid-derived suppressor cells (MDSCs), which contributed to nitric oxide (NO)-mediated tumor regression rather than suppressing viral spread [108]. Reprogramming the TME using chemokine-modulating (CKM) therapies has shown the potential to improve VV-based immunotherapy. VV can also be used as a vector to deliver immunostimulatory cytokines, amplifying systemic anti-tumor responses [109]. In summary, VV exerts cytolytic effects and expresses transgenes such as IL-12, IL-9, and checkpoint inhibitors. It prevents genome integration and operates in hypoxia.

#### 3.5.2. Clinical and Preclinical Evidence

Several engineered VV constructs have demonstrated potent cytolytic activity in CRC models. One example is a conditionally replicative VV (VV-FCU1), which encodes the suicide gene FCU1. This gene converts the prodrug 5-fluorocytosine (5-FC) into the chemotherapeutic agent 5-FU, resulting in significant tumor reduction in both subcutaneous and orthotopic liver metastasis models of human CRC [110].

Jeong et al. (2020) [111] developed a novel VV construct (NOV) co-expressing TRAIL and angiopoietin-1 (Ang1), replacing viral thymidine kinase (vTK) and vaccinia growth factor (VGF) to enhance tumor selectivity and reduce off-target effects. NOV exhibited superior oncolytic activity, apoptosis induction, and therapeutic efficacy compared to conventional VV strains. In syngeneic CRC mouse models, NOV significantly suppressed tumor growth, increased CD8^+^ T cell infiltration, and prolonged survival [111]. Additionally, VVs engineered with deletions of immunomodulatory genes such as N1L, K1L, K3L, A46R, and A52R have shown enhanced tumor selectivity and anti-tumor efficacy. These deletions reduce the virus’s ability to evade host interferon responses, thereby decreasing replication in normal tissues while maintaining infectivity in tumor cells. This approach improved CRC models’ tumor lysis, immune infiltration, and survival outcomes [112].

Histone deacetylase inhibitors (HDIs) are epigenetic modulators that suppress antiviral responses, particularly IFN signaling, making cancer cells more susceptible to viral infection [113,114]. Trichostatin A (TSA), a potent HDI, was found to significantly improve vaccinia virus (VV) replication and spread in cancer cells, while having minimal effects on normal cells [115]. Deleting the B18R gene, which encodes a soluble IFN receptor, enhances VV safety by increasing viral clearance from normal tissues, although it may also reduce viral replication in tumors [116,117]. In a colon cancer xenograft model, combining TSA and a B18R-deleted VV delayed tumor progression and increased survival compared to either treatment alone [118]. An oncolytic VV expressing an anti-CD47 nanobody (OVV-αCD47nb) blocks the CD47-SIRPα “don’t eat me” signal, enhancing macrophage-mediated phagocytosis and promoting CD8^+^ T cell activation. OVV-αCD47nb outperformed unmodified VV in tumor regression and survival, and reprogrammed tumor-associated macrophages from an M2 immunosuppressive phenotype to an M1 proinflammatory state [119]. On the other hand, combining vvDD-CXCL11 with CKM cocktails increased Th1-associated chemokines (CXCL9, CXCL10) and reduced Treg-attracting chemokines (CCL22, CXCL12), enhancing immune cell recruitment and survival in CRC models [120]. Similarly, VV engineered to express the chemokine CCL19 promoted the infiltration of dendritic cells and CD4^+^ T cells while maintaining effective viral oncolysis [121].

Chen et al. (2019) [122] developed a VV encoding interleukin-23 (IL-23), which enhanced immune cell infiltration, promoted Th1 chemokine production, and transformed immunologically “cold” tumors into “hot” tumors. These effects were mediated through IL-10 upregulation and required CD4^+^ and CD8^+^ T cells and IFN-γ for therapeutic efficacy. In CRC models, IL-9 expression increased viral persistence, modulated the TME by reducing MDSCs, and increased the infiltration of both CD4^+^ and CD8^+^ T cells. Combined with anti–CTLA–4 therapy, this strategy significantly improved anti-tumor immunity and survival [123,124]. Combinatorial therapies involving VV and immune checkpoint blockade have also shown promise. Co-administration of VV with anti–PD-L1 therapy led to stronger CD8^+^ T cell activation, reduced T cell exhaustion, and improved immune responses. This combination reduced metastases and prolonged survival in colon tumor-bearing mice compared to monotherapy [125].

Further enhancement was observed using recombinant VV expressing interleukin-15 (IL-15) and its receptor subunit IL-15Rα. This construct increased CD8^+^ T cell activity and cytokine release, leading to durable tumor regression. Combined with anti-PD-1 therapy, complete tumor eradication and long-term survival were achieved in all treated mice [126]. Similarly, VV expressing tethered IL-12 has been shown to promote CD8+ T cell infiltration, reduce Tregs, and transform cold tumors into hot tumors, improving survival. Combined with PD-1 blockade, it resulted in complete tumor regression in late-stage tumors [127].

### 3.6. Measles Virus (MV)

#### 3.6.1. Mechanism of Action

Oncolytic MV, derived from the attenuated Edmonston B strain, has shown promise in preclinical models of CRC due to its natural tumor tropism and immunogenicity [128]. Recent genetic modifications have further enhanced its tumor selectivity, immune-stimulating capacity, and safety profile, making MV an attractive candidate for virotherapy in CRC. MV selectively infects cells overexpressing CD133 or uPAR and induces strong immune responses, especially when armed with IL-12 [129].

#### 3.6.2. Clinical and Preclinical Evidence

Wang et al. (2021) explored the anti-tumor efficacy of an oncolytic measles virus encoding interleukin-12 (MeVac FmIL-12) in colon cancer [130]. In both in vivo and ex vivo models, MeVac FmIL-12 significantly enhanced anti-tumor immunity by upregulating proinflammatory cytokines, promoting apoptosis, and reducing cancer cell viability. This immune activation translated into systemic tumor rejection and prolonged survival in a rat model, demonstrating the potential of IL-12-armed MV as an immunovirotherapy platform for CRC [130].

Jing et al. (2009) [131] developed a retargeted MV that explicitly recognizes the urokinase-type plasminogen activator receptor (uPAR), a surface protein commonly overexpressed in solid tumors, including colon cancer. The modified virus efficiently infected and lysed uPAR-expressing tumor cells, inhibited tumor progression, and extended survival in preclinical models. Systemic administration facilitated targeted tumor tissue and vasculature infection, supporting its use as a tumor-specific oncolytic vector [131]. Further safety and efficacy evaluations of this uPAR-targeted measles virus (MV-m-uPA) were conducted by Jing et al. (2014) in syngeneic colon and mammary tumor models [132]. MV-m-uPA selectively infected uPAR-overexpressing CT-26 colon cancer cells, induced apoptosis, and suppressed tumor growth while prolonging survival. Notably, the virus exhibited preferential tumor accumulation without causing systemic toxicity, reinforcing its safety for future translational applications in CRC [132].

In another approach targeting tumor-initiating cells (TICs), Bach et al. (2013) [133] engineered CD133-specific oncolytic measles viruses to eliminate this stem-like subpopulation in solid tumors, including CRC. These CD133-targeted viruses exhibited enhanced infection and cytolytic activity against CD133^+^ tumor cells, outperforming non-targeted MV strains. Table 1 summarizes the clinical trials conducted with each oncolytic virus in CRC. Table 2 is a comparative table of the OVs used in CRC, considering the genome, tumor selectivity, immunostimulatory potential, clinical safety, clinical maturity, key advantages, and critical limitations.

## 4. Selection Criteria for CRC Patients for Trials with OVs

In clinical trials evaluating oncolytic adenovirus, participant selection exhibits similarities across the different studies. First, most trials include patients with a good performance status, as these individuals are more likely to tolerate immunotherapy interventions [134,135,136,137,138,139,140,141,142,143]. Additionally, eligibility generally requires adequate hematologic, hepatic, and renal function to minimize the potential risk of treatment-related toxicity. Conversely, patients who are commonly excluded are immunocompromised patients or those with active autoimmune disease, and pregnant or breastfeeding women. Secondly, some differences observed in the studies are related to disease status. While some studies enroll patients with metastatic or active tumors, others are restricted to individuals with resected tumors or patients with no evidence of disease after resection (NED). Interestingly, some trials exclude patients harboring molecular biomarkers such as MMR-D/MSI-H, who would respond better to standard immunotherapy [137]. In contrast, some studies include tumors with antigen overexpression, such as CEA, MUC1, and GCC, which represent potential vaccine immune target proteins [138,139,140,141]. In terms of the population characteristics, the Ad5-hGCC trial includes Caucasian and African American colon cancer patients in stages I and II as participants, unlike the rest of the trials that do not specify ethnic origin [140]. Furthermore, studies such as the IL-2 trial incorporate tumor burden as part of their criteria; however, other metastatic CRC trials do not include tumor volume as a selection factor [139].

In the context of oncolytic herpes virotherapy, participation criteria share several similarities with those observed in adenovirus-based therapy. However, unlike adenovirus trials, most herpes-based studies are focused on advanced, metastatic, and refractory tumors [144,145,146,147,148]. Regarding the tumor burden, both the RP2/RP3 and ONCR-177 trials require the presence of hepatic metastasis and at least one injectable tumor(s) ≥1 cm [144,145,146,147,148]. The OH2 trial, in contrast, includes stable post-chemotherapy and accessible lesions [147]; on the other hand, the NV1020 trial excludes patients with extrahepatic disease [146]. In terms of molecular biomarkers, RP2/RP3 is the only trial among those reviewed that excludes MSI-H and BRAF V600E mutations as part of the selection criteria [144]. Furthermore, most trials require at least four weeks to have elapsed since the last immune and chemotherapy treatment as a restriction on previous therapies [144,145,146,147,148]. Finally, both NV1020 and ONCR-177 protocols prohibit the concomitant use of antiviral therapy against HSV, due to the potential interference with virus replication [146,148].

Regarding vaccinia virotherapy, the clinical status of cancer at the time of participant selection varies across trials. Most enrolled patients exhibited tumoral progression despite multiple lines of treatment, typically characterized by disseminated and unresectable disease [149,150,151,152,153,154,155,156,157]. In contrast, studies such as CV301 + nivolumab or vaccinia-CEA-TRICOM + docetaxel include patients with resectable metastases, who are generally treated with curative intention and have no evident disease [150,156]. Another difference between trials is the selection of specific biomarkers. For instance, the PEXA-VEC + ICI (JX-594) trial actively includes MSS tumors (and excludes those with MSI-H) because these tumors are unlikely to respond to standard ICI therapy [155]. Similarly, the p53MVA and p53MVA-plus-pembrolizumab trials include p53-overexpressing cancers as part of the target protein of the viral vector [151,152]. Additionally, vaccinia-CEA-TRICOM + docetaxel and vaccinia-CEA-MUC-1-TRICOM incorporate the biomarker CEA, a tumor-associated receptor recognized by TRICOM-based vaccines [156,157]. Notably, the vaccinia-CEA-TRICOM + docetaxel trial specifies that at least 6 of 10 patients in each treatment arm must be HLA-A2 positive [156]. Lastly, one of the JX-594 trials uses KRAS/EGFR status to determine eligibility after anti-EGFR treatment failure (if KRAS WT) [154].

## 5. Limitations, Challenges, and Future Perspectives

### 5.1. Mechanisms of Resistance

Despite substantial advances in oncolytic virotherapy, multiple resistance mechanisms that limit viral replication, tumor cell killing, and systemic immune activation in CRC models have been identified. One such mechanism involves host-derived antimicrobial peptides. Human α-defensin 5 (HD5), secreted by epithelial cells, has been shown to interfere with oncolytic adenovirus therapy by binding to viral capsid proteins, thereby blocking endosomal escape and viral replication [158,159,160]. Interestingly, human adenovirus serotype 3 (HAdV3) can partially evade this inhibition by producing penton-dodecahedral (PtDd) particles that neutralize HD5, allowing for continued viral dissemination [160]. These findings suggest that tumor-expressed HD5 represents a significant barrier to adenovirus-based therapies, necessitating the design of viral vectors capable of overcoming such innate defenses.

Interactions between chemotherapeutics and virotherapy can also compromise viral replication. Kulu et al. (2013) [161] demonstrated that 5-FU and irinotecan (CPT-11) inhibit HSV-1 replication in colon cancer cells by disrupting NF-κB signaling and phosphorylating eIF-2α. Since NF-κB activation is essential for HSV-1 propagation, chemotherapy-induced signaling events may create a cellular environment that is unfavorable for viral replication, highlighting the importance of optimizing therapeutic combinations [161].

Resistance to oHSV-1 therapy has also been linked to gene expression changes that impair viral entry, replication, and induction of apoptosis. Studies using HSV-1-resistant CRC cell lines revealed alterations in surface receptors, metabolic pathways, and immune signaling components contributing to therapy failure [162].

Reovirus therapy, similarly, faces limitations. Vanhoudt et al. (2008) [163] reported that intact reovirus T3D virions failed to infect freshly isolated human colorectal tumor cells, regardless of KRAS mutation status. Only intermediate subviral particles achieved transient infection, producing viral proteins without generating infectious progeny or inducing cytolysis. This resistance was attributed to the abnormal localization of the junctional adhesion molecule 1 (JAM1), the primary reovirus receptor, and cellular responses that abort viral replication [163].

Resistance to VSV is frequently driven by the chronic activation of innate immune pathways. Transcriptomic profiling identified the elevated expression of interferon-stimulated genes (ISGs), including Mx2, Oasl, and Irf7, which conferred cross-resistance to both VSV and Sindbis viruses [164]. These resistance signatures were conserved across diverse tumor cell lines, indicating a reproducible antiviral state. Additional studies in CT26 colon carcinoma cells revealed heterogeneous resistance mechanisms, including one clone characterized by immune activation and ISG expression and another defined by cytoskeletal remodeling and altered intracellular signaling [99]. Together, these outcomes underline the need for strategies to counteract innate immune activation to enhance the efficacy of oncolytic virotherapy.

The inhibition of viral replication also represents a critical challenge. The c-Jun N-terminal kinase (JNK) pathway, known to regulate stress responses and apoptosis, can limit viral propagation. JNK-deficient cells showed a 100-fold increase in VV replication and virus-induced apoptosis, suggesting that JNK acts as a viral restriction factor, likely by activating the double-stranded RNA-dependent PKR pathway [165,166,167,168,169]. Therefore, modulating stress-related kinases may improve the replication and efficacy of oncolytic viruses.

### 5.2. Personalized Strategies and Combined Therapies

Numerous innovative strategies are being developed to overcome the limitations of single-agent virotherapy, including personalized vaccines, combinatorial virus designs, and the modulation of host immune responses.

Feola et al. (2022) [170] developed a personalized immunopeptidomics-based approach to generate oncolytic vaccines tailored to colorectal tumors. Their platform, PeptiCRAd, integrates oncolytic adenoviruses with tumor-specific MHC-I-restricted peptides, enhancing antigen-specific immune responses and tumor suppression in preclinical models [170]. The same group created PeptiCab, a novel adenovirus-based cancer vaccine targeting Fcγ and Fcα receptors to amplify adaptive immunity. This construct incorporates tumor antigens and a PD-L1 inhibitor, promoting neutrophil polarization toward an antigen-presenting phenotype and boosting T-cell responses [171]. A second approach involves enhancing the fusogenic potential of oncolytic viruses. VSV-p14, a recombinant VSV expressing the reovirus-derived fusion-associated small transmembrane (FAST) protein p14, demonstrated improved tumor cell fusion, viral spread, and immune activation. In CT26 tumor models, VSV-p14 significantly increased CD4^+^, CD8^+^, NK, and NKT cell infiltration, resulting in superior tumor control and prolonged survival [172].

Another strategy is the inactivation of key tumor resistance factors. As mentioned above, VSV is a promising oncolytic therapy for colon cancer, yet some tumors develop resistance [99,164]. Because of this, a study has identified Cancer Upregulated Gene 2 (CUG2) as a factor whereby tumor cells resist VSV infection through STAT1 activation and OASL2 upregulation, enhancing antiviral defenses. Suppressing STAT1 or OASL2 restored VSV susceptibility, increasing viral replication and apoptosis in resistant tumor cells. These results suggest that targeting CUG2-mediated pathways could improve VSV-based virotherapy’s efficacy in resistant CRC [173].

Another promising strategy involves engineering viruses with enhanced fusogenicity. Fusogenic vaccinia virus (FUVAC), which carries a mutation in the K2L gene to promote syncytia formation, demonstrated superior tumor cell killing, immunogenic cell death, and CD8^+^ T cell recruitment. In bilateral colon tumor models, FUVAC induced the regression of both treated and distant tumors. Combined with PD-1 blockade, FUVAC achieved complete tumor regression and long-term immune memory [174,175,176].

Additionally, inducing ferroptosis—a form of iron-dependent cell death—may potentiate oncolytic virotherapy. Co-treatment with elastin and VV led to reduced tumor growth, extended survival, and enhanced immune memory. Mechanistically, elastin promoted dendritic cell maturation and CD8^+^ T cell activation, increasing IFN-γ^+^ and PD-1^+^ T cells within the TME [177]. These results suggest that ferroptosis induction can synergize with VV to augment anti-tumor immunity in CRC.

### 5.3. Virotherapy’s Clinical Limitations in Colon Cancers

Despite the significant advances in virotherapy in CRC, several limitations are observed when put into clinical practice. One limitation in the clinical application is the acquisition of viral resistance. Cancer proteins responsible for suppressing cytokine signaling and members of the integrin class of cell surface receptors may develop resistance that reduces viral attachment and increases acute-phase responses against viral infection [162]. In the same way, antiviral antibodies are more likely to be found after repeated oncolytic measles virus exposure, suggesting a neutralizing activity under long-term treatments [132,178]. Physical barriers caused by cytoskeletal reorganization and poor vascular perfusion may impede viral diffusion to cancer cells and reduce therapeutic efficacy [99].

Another concern in the application of virotherapy is the risk of toxicity in healthy tissues. One example of this is the administration of vaccinia virus, which has shown no dose-limiting toxicities. However, researchers express doubts regarding patients without prior vaccinia vaccination and immunosuppressed patients, who were excluded from their studies [179]. Intriguingly, the persistent stimulation of the T cell immune response by viral therapy may turn into a dysfunctional or “exhausted” state, which alters treatment outcomes; however, combined strategies could synergistically enhance the anti-tumor adaptive immune response and reverse T cell exhaustion within the TME [84].

The systemic administration of viral therapy has also shown limitations, such as healthcare problems and immune sensitivity, resulting in undesirable side effects [180,181]. Therefore, researchers have developed local approaches, such as nanomaterials, to deliver viruses to the site of interest [182]. The intravenous infusion of virotherapy is a safe treatment delivery modality in metastatic CRC; however, this may also be challenging because it results in a less effective response than locoregional delivery due to systemic viral clearance [178].

Lastly, the use of virotherapy as monotherapy in colon cancer has shown limited clinical results due to the production of immunosuppressive factors for tumor protection. Thus, most current efforts focus on combining different strategies to enhance therapy efficacy while avoiding cell toxicity [183]. For instance, oncolytic reovirus that sensitizes microsatellite stable (MSS) CRC to immune checkpoint inhibition, such as anti-PD-1 treatment, increases cell death among MSS cells in mouse models. Although these findings shed light on promising combined immunological mechanisms in animal models, the authors also mention that translating preclinical findings to the clinic remains a significant challenge [84].

## 6. Conclusions

OVs represent a promising therapeutic approach for CRC that takes advantage of genetically modified viruses’ ability to selectively target and lyse tumor cells while stimulating anti-tumor immune responses. The efficacy of virotherapy is based on multiple mechanisms, including direct oncolysis, the modulation of the TME, and the enhancement of systemic immune responses. Despite these advances, several challenges hinder the widespread clinical application of OVs in CRC.

One major limitation is the development of resistance mechanisms that impair viral infection, replication, and immune activation. Tumor cells can evade virotherapy by secreting immunosuppressive factors, modifying cell surface receptors to prevent viral entry, and activating antiviral signaling pathways, including interferon-stimulated genes. Additionally, the TME can promote resistance by increasing regulatory T cells, myeloid-derived suppressor cells, and hypoxia, inhibiting the efficacy of OVs.

To overcome these challenges, novel strategies are being explored, including the genetic engineering of OVs to enhance tumor selectivity and immune stimulation. Combining virotherapy with immune checkpoint inhibitors, CAR-T cell therapy, and personalized cancer vaccines has significant potential to improve treatment outcomes. Additionally, fusogenic viruses, the modulation of tumor metabolic pathways, and the inhibition of antiviral defenses are emerging approaches for enhancing OV efficacy.

Future research should optimize OV delivery, reduce resistance mechanisms, and improve patient stratification to maximize therapeutic benefits. By addressing these limitations, oncolytic virotherapy could become a key component of multimodal treatment strategies for CRC, providing a more effective and personalized approach to cancer therapy.

## Figures and Tables

**Table 1:** Clinical trials of oncolytic virus therapy in patients with CRC *.

Virus Type	Therapy	Cancer Status	Study Status	Phase	Participants	Clinical Trial ID
Adenovirus	ColoAd1	Resectable	Completed	1	17	NCT02053220
	BioTTT001 + Toraplizumab and Regorafenib	Liver Metastasis	Not yet recruiting	1	40	NCT06283134
	GVAX	Liver Metastasis	Terminated	1 (Pilot study)	1	NCT01952730 ^†^
	Ad-CEA + avelumab	Metastatic	Terminated	2	30	NCT03050814 ^†^
	ETBX-011, ETBX-061, ETBX-051	Advanced	Completed	1	11	NCT03384316 ^†^
	IL-12	Metastatic	Terminated	1	22	NCT00072098
	Ad5-hGCC-PADRE	Stage I/II	Completed	1	1	NCT01972737
	Ad5.F35-hGCC-PADRE	Stage III/IV	Active, not recruiting	2	81	NCT04111172
	Ad-sig-hMUC-1/ecdCD40L	Recurrent or Metastatic	Unknown status	1	24	NCT02140996
	VB-111 + Nivolumab	Metastatic	Completed	2	14	NCT04166383
Herpes Simplex Virus	RP2/RP3 + Atezolizumab and Bevacizumab	Advanced	Active, not recruiting	2	4	NCT05733611
	T3011 + Toripalimab and Regorafenib	Liver Metastasis	Not yet recruiting	1	8	NCT06283303
	NV1020	Liver Metastasis	Completed	1	No data	NCT00012155
	OH2 + Capecitabine	Advanced	Terminated	2	7	NCT05648006
	ONCR-177	Refractory, Metastatic	Terminated	1	66	NCT04348916
Reovirus	REOLYSIN + FOLFIRI and bevacizumab	Metastatic	Completed	1	36	NCT01274624
Vaccinia Virus	GC001	Advanced	Recruiting	1	21	NCT06508307
	CV301 + Nivolumab and Systemic Chemotherapy	Metastatic	Active, not recruiting	2	78	NCT03547999
	p53MVA	Unresectable and chemotherapy resistant	Completed	1	12	NCT01191684
	p53MVA + pembrolizumab	Advanced	Active, not recruiting	1	11	NCT02432963
	JX-594	Refractory	Completed	1	15	NCT01469611
	JX-594	Liver Metastasis	Terminated	2a	2	NCT01329809
	JX-594	Metastatic, Refractory	Completed	1b	15	NCT01380600
	JX-594	Metastatic, Refractory	Completed	1/2a	52	NCT01394939
	JX-594 + Tremelimumab/Durvalumab	Refractory	Completed	1/2	34	NCT03206073 ^†^
	vaccinia-CEA-TRICOM + docetaxel	Metastatic	Terminated	1	60	NCT00088933 ^†^
	vaccinia-CEA-MUC-1-TRICOM	Metastatic	Completed	2	74	NCT00103142 ^†^
Measles Virus	MVF-HER-2 (266–296/597–626)	Metastatic	Completed	1	65	NCT01376505
	PD1-Vaxx	Operable high MSI ^a^	Not yet recruiting	2	44	NCT06692959

* Data taken from clinicaltrials.gov; neither suspended nor withdrawn from trials. ^†^ Results already published. ^a^ Microsatellite instability—high.

**Table 2:** Comparative analysis of OVs used in CRC.

Virus	Genome/Type	Tumor Selectivity	Immunostimulatory Potential	Clinical Safety	Clinical Maturity *	Key Advantages	Critical Limitations
Adenovirus	dsDNA, non-enveloped	High (e.g., LOI, p53 loss)	High (e.g., IL-15, GM-CSF)	Well characterized	Phase I–II in CRC	Genetically tractable, scalable production	Pre-existing immunity, blocked by HD5, limited systemic delivery
Herpes Simplex Virus (HSV)	dsDNA, enveloped	High (solid tumors)	Very high	High (e.g., T-VEC approved)	Phase I–II in CRC	Large transgene capacity, antiviral control options	Complex manufacturing, latency potential
Reovirus	dsRNA, non-enveloped	Moderate (RAS-mutant preference)	Moderate	Excellent	Phase I in CRC	Oral delivery, native tropism, minimal engineering	JAM1 receptor localization limits infectivity in primary tumors
Vesicular Stomatitis Virus (VSV)	ssRNA, enveloped	High (IFN-defective cells)	High	Moderate	Preclinical/Phase I	Rapid replication, fusogenic potential, and low seroprevalence	Systemic toxicity, fast immune clearance
Vaccinia Virus (VV)	dsDNA, enveloped	High (hypoxia adapted)	High	High (extensive safety data)	Phase I–II in CRC	Cytoplasmic replication, large genome for transgenes	Manufacturing burden, proinflammatory, and immune clearance
Measles Virus (MV)	ssRNA, enveloped	High (via uPAR/CD133 targeting)	High	High (attenuated strains)	Phase I in CRC	Tumor tropism, engineered targeting, systemic administration	Risk of neutralizing antibodies, less clinical experience in CRC

* Data taken from: clinicaltrials.gov.

## Glossary

OV	Oncolytic Virus
CRC	Colorectal Cancer
HSV	Herpes Simplex Virus
VSV	Vesicular Stomatitis Virus
VV	Vaccinia Virus
MV	Measles Virus
TME	Tumor Microenvironment
IL	Interleukin
GM-CSF	Granulocyte-Macrophage Colony-Stimulating Factor
TRAIL	TNF-Related Apoptosis-Inducing Ligand
aPD-1	Anti-Programmed Death-1
CXCL11	C-X-C Motif Chemokine Ligand 11
TLR3	Toll-Like Receptor 3
NK	Natural Killer (cell)
MDSC	Myeloid-Derived Suppressor Cell
Treg	Regulatory T Cell
ICIs	Immune Checkpoint Inhibitors
HDAC	Histone Deacetylase
CSC	Cancer Stem Cell
CAR-T	Chimeric Antigen Receptor T Cell
